# The concept of the contact angle in the process of oil film formation in internal combustion piston engines

**DOI:** 10.1038/s41598-023-47763-9

**Published:** 2023-11-24

**Authors:** Piotr Wróblewski, Stanisław Kachel

**Affiliations:** 1 Faculty of Engineering, University of Technology and Economics H. Chodkowska in Warsaw, Jutrzenki 135, 02-231 Warsaw, Poland; 2 Faculty of Mechatronics, Armament and Aerospace of the Military University of Technology, ul. gen. Sylwestra Kaliskiego 2, 00-908 Warsaw 46, Poland

**Keywords:** Materials science, Engineering, Mechanical engineering

## Abstract

In internal combustion piston engines, the process of oil film formation differs from that in industrial machines. The adhesive strength of the molecules at the interface between the coating of engine parts and the lubricating oil affects the load carrying capacity and the ability to form a lubricated film. The geometry of the lubricating wedge between the surfaces of the piston rings and the cylinder wall is determined by the thickness of the oil film and the amount of oil coverage of the ring. This state is modified by many parameters describing the operation of the engine and the physical and chemical parameters of the coatings of the cooperating pairs. For lubricating molecules reaching energies greater than the energy barrier of adhesion at the boundary, sliding occurs. Therefore, the value of the contact angle of the liquid on the surface of the coating depends on the value of the intermolecular force of attraction. According to the author, there is a strong correlation between the contact angle and the lubrication effect. Research indicates that the potential barrier is a function of the contact angle and the contact angle hysteresis (CAH). The innovation of the work consists in the study of the contact angle and CAH in the conditions of thin layers of lubricating oil in cooperation with hydrophilic and hydrophobic coatings. The thickness of the lubricating film was measured under various speed and load conditions using optical interferometry. The study proves that CAH is a better interface parameter to correlate with the effect of hydrodynamic lubrication.

## Introduction

The surface characteristics of materials are of key importance in various chemical, physical and biological processes^1^. Hydrophobicity is one of the dominant surface features that is shaped by the wetting ability, determined by the contact angle (CA) measured using the resting drop technique or the sitting technique^2^. Superhydrophobic surfaces are characterized by extremely low wetting capacity (low surface wetability), and such features are found in nature. These surfaces have water contact angles greater than 150° and low dip angles of less than 10°. They are achieved by reducing surface energy and creating hierarchical/micro-nanostructures^3–5^. Superhydrophobic biological surfaces in nature, such as lotus leaf, rose leaf, water strider, butterfly, etc., are not wetted by rain^6,7^. Superhydrophobic surfaces have attracted great interest for over a decade biomedical, self-cleaning, anti-biofilm, oil-water separation, anti-corrosion and anti-freeze applications, among others^8–13^.

In recent years, many methodologies have been developed to produce hydrophobic surfaces on a variety of substrates such as polymeric, silicon, metallic, composite and alloy^14^. These strategies involve the use of chemical reagents (e.g. fluoroalkylsilanes) to reduce surface energy and the creation of hierarchically ordered surface forms (micro/nano-forms) to enhance surface roughness, thus imitating natural mechanisms^15^. Techniques used to achieve superhydrophobicity include the sol-gel process^16^, anodizing^17^, lithographic methods^18^, chemical vapor deposition^19^, photolithographic methods^20^, electrodeposition processes^21^, and etching chemical and plasma^22^, among many others. Standing out among them, laser texturing, which is a direct maskless technique, presents a number of advantages such as minimal thermal deformation of the substrate, versatility, ability to create complex geometries, no need to contact the material, simplicity and eco-friendliness^23,24^. Nanostalactites, nanoreliefs and nanopaths are examples of forms obtained on substrates during the laser texturing process^25^. Numerous studies have confirmed that laser-treated surfaces are initially hydrophilic, but at a later stage evolve towards superhydrophobicity after exposure to the surrounding atmosphere, treatment with oxygen-rich plasma or annealing at reduced temperature^26–28^.

In internal combustion piston engines, there are changes in the hydrodynamic pressure in the engine's work cycle. They especially affect the coating of the piston, piston rings and cylinder wall with an oil film. The value of these changes depends on many geometrical parameters of engine structural elements, its operating conditions and chemical parameters of the lubricating fluid. The surface properties of the material, mainly hardness, roughness, Young's modulus and surface energy, also have a significant impact on the distribution of the oil film. In the literature, many researchers focus on designing the surface topography, ignoring such an important parameter as the hydrophilic and hydrophobic properties of coatings. As the author's previous works indicate, the influence of these parameters is very important and should not be overlooked^29^. Contact angle hysteresis (CAH) is one of the most important and classic elements of fluid droplet wetting in tribological systems. There are many papers devoted to the definition of CAH^30–32^. According to studies^33–36^, it is recognized that accurate contact angle measurements can be used to calculate surface tension constants. In recent years, numerous methods have been used to measure the contact angle^37–40^. The most important surface parameters are: self-cleaning, adhesion, low friction, wettability, hydrophobicity and/or hydrophilicity and surface energy. They are of great importance for the properties of coatings and base materials as well as lubricants. In most works on the tribological behavior of cooperating pairs in internal combustion engines and industrial machines, these parameters are largely omitted. They are not fully taken into account in most theoretical works and simulation models. It should be added that the parameters of coatings used in combustion engines, such as surface energy, microgeometry, hydrophilicity, hydrophobicity, contact angle, contact angle hysteresis, have been described only in a few articles. In addition, it is worth paying attention to the developing technologies of coatings that can significantly affect the tribological properties, such as nanotechnology or diamond-like coatings, which show high hardness and low roughness, which is conducive to better distribution of the oil film.

The results and conclusions of the conducted research are varied, which makes it difficult to assess their credibility on the theoretical level^41–43^. Experimental comparisons were also made, but under different conditions—both on the macro scale and under hydrodynamic conditions or contacts on the nano scale^44,45^. Macro contact studies show more consistent results^46–51^. An additional difficulty in the context of engineering models is the fact that experiments are carried out in conditions that do not correspond to reality, for example on model surfaces or with the use of model fluids^52–54^. Experiments using surfaces similar to those used in internal combustion engines and oils have been presented in Refs.^55–57^. Despite the growing number of scientific works in the field of wetting, there is still a lack of full understanding of the phenomena occurring in internal combustion engines. Missing models and miscellaneous theories often disagree, leading to large discrepancies in research results. This often applies to mapping the stereometry of surfaces of cooperating elements, kinematics and dynamics of elements operating in the main mechanism and engine timing. Inaccuracies in the description of the flow of gases and fluids, the lack of appropriate input data reflecting the operating conditions of the engine, and in particular the heat load resulting from the thermodynamics of combustion of the fuel-air mixture are common. Currently, there are almost no scientific papers on simulation models of cooperation of pistons and piston rings with the cylinder surface, taking into account data on wettability, hydrophilic and hydrophobic properties, full surface stereometry and the phenomena of simultaneous flow of liquid and gas through nanospaces of friction pairs. Tests using a real engine were carried out by the author for the first time and described in detail in Ref.^29^. According to the author the determination and control of CAH is critical to the functioning of these tribological systems. It is worth noting that not only experimental conditions, but also measurement tools and technologies are evolving, enabling more accurate and representative research. Modern technologies enable analysis at the nano and micro level, which can significantly affect the accuracy and reliability of the results. Further research in this area is needed to better understand the complex interactions in internal combustion engines and adapt theoretical models to reality.

Many experimenters have conducted research on lubrication theory using CAH. With their help, it is possible to understand certain mechanisms of operation of this theory in the context of internal combustion engines. Molecular dynamics (MDS) simulations were performed in Ref.^42^. It was found that the slip value increases with the greater coating wettability angle. The slip length reaches up to thirty molecular diameters, which takes place at a contact angle of about 140°. Using the MDS for the water model, the wettability angle defines the slip control^43^. In the assumption of the study, it was assumed that the exponential characteristic defining the relationship between the slip length and the value of the wettability angle for fluid-repelling surfaces represents the growth rate of the slip length. In another paper^44^, measurements of the liquid penetration force between the sphere and the hydrophilic and hydrophobic coating were carried out. The penetrant was glycerol and the measurements were made using a measuring apparatus surface forces (SFA). Slip occurred only in non-wet conditions. By measuring the speed of particles, it is also possible to identify the occurrence of limit slip^55^. In this work, the apparent speed of sliding as water flows through a hydrophobic channel was noted. In Ref.^56^, a dynamic surface force apparatus was used to measure interaction forces in water. The authors of this paper observed that water slippage increases with hydrophobicity. Studies on the slip and its length in a Newtonian fluid using the SFA method were carried out in Ref.^57^. With regard to kinematic couples hydrodynamically lubricated at low loads, studies have been carried out in Refs.^58,59^. Glycerol and n-hexadecane lubricants were used. To a large extent, most of the research on surface-liquid interactions deals with the measurement of drainage or hydrodynamic forces. A comparison of the ability to form a lubricating film for different surfaces is presented in Ref.^60^. Interferometry was used to accurately measure at the micro scale. As part of these measurements, it was found that a surface with high wettability and a small contact angle produced a greater thickness of the lubricating film. This is due to the intermolecular attraction of the lubricating liquid to the surface layer of the coating. The results were inconclusive. In works^61,62^ different drainage forces were obtained for the flow of water on a fully wetted tribological system. In the work^63^ it was found that the coefficient of friction under EHL lubrication conditions does not depend on the hydrophobicity of the coating. The test was performed on a PDMS target with a bullet. Nevertheless, the results showed that in the boundary lubrication regime, the coefficient of friction decreases as the contact angle decreases. In the work^64^, the hydrodynamic force was measured in various liquids with the surface covered with alkylsilane and the colloidal AFM probe.

It should be noted that the lubricant flow in labyrinth seals or bearings is dynamic, while the contact angle is a static parameter reflecting the range of wettability. A variety of conclusions regarding the influence of wettability were drawn in these studies. In many of them, it was observed that the dynamics of the lubricant flow in various systems is crucial for the functioning of mechanisms, especially in the context of contact angle and wettability. Nevertheless, it must not be forgotten that each surface tested and each fluid used has its own unique characteristics that may affect the observed results. This issue is extremely important in the context of engineering, where a thorough understanding of these phenomena can be applied to the practical design and optimization of systems. Therefore, it is important to conduct further research in this area in the future, which takes into account the diversity of experimental conditions and materials. In some of the cited works^65,66^ it has also been suggested that certain surface parameters, such as roughness or microstructure, may play an equally important, if not more important, role in the dynamics of fluid flow in tribological systems. The use of modern measurement technologies, such as the atomic force microscope (AFM) or spectroscopy, may in the future provide more in-depth knowledge on this subject. In addition, the evolution of materials used in industry must not be forgotten, which can significantly affect the understanding of the discussed phenomena. For this reason, it is important to conduct research not only on traditional materials, but also on modern composites and nanomaterials that may have unique tribological properties. In the context of all the work discussed, it is worth emphasizing that although many important discoveries have been made, there are still many unknowns in the field of lubrication theory, wettability and tribology. The introduction of new research methods and an interdisciplinary approach to the problem can bring a breakthrough in this field in the near future.

The aim of this paper is to analyze and compare the effectiveness of the contact angle and the hysteria of the contact angle (CAH) in correlation with hydrodynamic lubrication, based on the example of a labyrinth sealer. Standard coatings and lubricating oils used in engines were used for this. Importantly, the use of modern technologies in the creation of coatings and additives for lubricating oils, which may be the future in terms of reducing friction losses in internal combustion engines, has been indicated. The key innovation of this study is the application of traditional measurement techniques to new material technologies, allowing for a more in-depth understanding of the complex interactions that occur at the interface between coating and lubricating oil. Thanks to this, it is possible not only to understand how different surface parameters affect the lubrication processes, but also how they can be optimized to obtain the desired tribological properties. The author emphasizes that these results are gaining full significance only when compared with the results of tribological tests and tests on engine benches. Thanks to this, it is possible to obtain a more complete picture of the actual behavior of coatings and oils in conditions close to real. The results of this work indicate the essence of the problem of hydrophilic and hydrophobic interactions on the process of oil film decomposition. Using modern technologies, theory and practice can be combined, creating new opportunities for engineers and researchers in their quest to achieve optimal lubricating properties. In the era of growing ecological interest and the search for ways to increase energy efficiency, such research is extremely important. Overall, the presented research provides important information on the interactions between surfaces and fluids in the context of hydrodynamic lubrication. They indicate potential directions of development in the field of designing new coatings and additives for oils lubricants that can help to increase the efficiency and durability of internal combustion engines.

## Materials and methods

To conduct a series of tests, a specialized optical station was built to test sliders with a constant slope, based on the studies described in Ref.^65^. Sliding friction is achieved by a glass disk rotating on a stationary inclined slider. The angle of inclination can be adjusted freely, adapting to the expected results. The thickness of the oil film is measured by interferometry. In order to support the initial reflection of the incident ray at the liquid-solid interface, the glass surface was covered with a layer of sprayed chrome. As the disc rotates, the hydrodynamic effect causes the slider to lift. During the test, the number of interference fringes is identified to determine the slope. When the test is turned off, the slider falls freely to the disk surface. The exact determination of the film thickness for a specific reference speed, in accordance with the shift of kinematic pairs in the internal combustion engine, is based on the change in the order of the fringes and the intensity of a selected point of the interferogram during the hold period with the multi-beam intensity approach^66^. In a piston internal combustion engine, the average thickness of the oil film under load conditions, e.g. during the expansion stroke, does not exceed 10 μm. This maximum value was adopted in experimental studies. Measurement uncertainty for this stand was less than 8 nm. In addition, it is worth noting that such research is crucial in the context of improving the performance and durability of engine components, as precise understanding and control of oil film thickness can significantly reduce wear and friction in these key engine components. Modern research methods, such as the described interferometry, allow for more and more accurate and representative modeling and analysis of the behavior of lubricating films in real operating conditions.

In the experimental analysis, a glass disc covered with a layer of chromium and SiO2 (silicon dioxide) was used. This specific configuration was to ensure appropriate resolution conditions for the optical system and to protect the surface from damage or degradation. It is worth noting that the reflectivity of the chrome coating is about 20%, which is crucial for maintaining the optical properties of the experiment. During the test, 5 different coatings were applied to the slider of the device. The dimensions of the sliding plane are 5.15 mm by 10.20 mm. Material properties and their roughness are presented in Table 1. Steel intended for the production of rings (slider 1) was used as the basic material in the test. It is stainless steel 1.4112 (X90CrMoV18) with high hardness and abrasion resistance. Various multi-layer and single-layer nanocoatings (sliders 2–5) were applied to this steel. Hydrophobic and hydrophilic coatings are adopted. All samples had roughness measured in nanometers, co confirms the precision of the process and the uniformity of coverage. Three lubricants were used in the study: glycerol with a content of 65 wt.%, glycerol with a content of 99 wt. and lubricating oil. The properties of these greases are shown in Table 2. Contact angle and CAH were measured in a test of these lubricants on the surfaces of accepted specimens. Depending on the sample, the liquid drop needed a short exposure time to stabilize the drop's optical parameters. It lasted a maximum of 10 seconds. The measurement was considered valid after stabilization. The test was performed 5 times with the same fluid and sample. The measurement error was not greater than 3.5% for all samples and lubricants. Before testing, all samples were washed in an ultrasonic bath in 99% alcohol. The impurities were removed with a special cloth that absorbs liquid and solid impurities. The slider was dried for about 3 minutes. In addition, with such advanced research, important is the control of environmental conditions, such as temperature or humidity, which can affect the measurement results, especially with such high precision. It is also worth noting that the accuracy of the measurements, as well as the cleaning of the samples, are crucial to obtain reliable and reproducible results in such experiments.

**Table 1:** Properties of kinematic pairs of the interferometric study.

Slider type	Surface layer	Bulk material	Roughness (nm)	The number of layers
1	Steel X90CrMoV18	Steel X90CrMoV18	123±3	0
2	AlTiN/CrN/Cr/…CrN/Cr	Steel X90CrMoV18	121 ±4	1–AlTiN, 5–CrN, 5–Cr
3	AlN/CrN/…/AlN/CrN	Steel X90CrMoV18	108+3	6–AlN, 5–CrN
4	CrN/AlN/…/CrN/AlN	Steel X90CrMoV18	139±4	5–CrN, 5–AlN
5	Glass	Cr	4±0.3	1–Cr
6	Glass	SiO_2_	5±0.4	1–SiO_2_

**Table 2:** Parameters of lubricants.

Lubricant	Refractive index	Dynamic viscosity (22 °C, mPas)
Oil	1.46	840
99 % Glycerol	1.47	704
65 % Glycerol	1.45	14

Table 3 summarizes the results grouped by different fluids and base materials. Under ideal conditions, based on Young's equations^67^, one would expect a unique contact angle of a liquid droplet on a perfectly smooth and flat solid surface. Under experimental test conditions, a range of contact angles can be measured. The upper and lower limits of the range are determined by the increase or decrease of the contact angles. The CAH contact angle hysteria can be represented as the difference between the increasing and decreasing CA contact angle. The studies used the sedentary method to determine CAH. A drop of test liquid with a volume of less than 10 μl was applied to the surface layer of the steel or coated steel sample. Liquid was added to the drop until the line of contact moved. At the appropriate time, a measurement was made for the advancing contact angle. Alternatively, liquid was taken from the drop. At the time of retreat, the angle of contact was reached as the line of contact was moving. Tests conducted at an ambient temperature of 17.0 ± 0.5 °C and humidity of 30 ± 1%. A new glycerol was used for each study group. It is well known that glycerol is hygroscopic. Therefore, the time change in the viscosity of the 99 wt% glycerol solution was determined. In the test area, its viscosity was assumed to drop by only 1.1% in 20 minutes. The viscosity of all fluid samples was constant during the test. No changes in viscosity greater than 0.1% were found, therefore it was assumed that the viscosity of the fluid samples did not change. The materials adopted were characterized by divergent contact angle properties with similar material properties. Some of the materials are described in publications^28,68,69^. In other experiments of this type, various fluids and base materials were analyzed for their ability to create specific contact angles. In one study, described in publication^70^, different base oils were used and contact angles were measured on surfaces with different nanoparticulate coatings. Results showed that coatings based on zinc oxide nanoparticles gave lower contact angles compared to titanium oxide coatings. In another study^71^, various aqueous solutions were used and it was found that the addition of salt significantly affects the contact angle, especially in the case of hydrophobic surfaces.

**Table 3:** Analysis of the contact angle value and contact angle hysteresis for various surfaces and liquids.

Lubricating liquids	Zipper surface material	Contact angle CA (°)	Contact angle hysteresis CAH (°)
65 % Glycerol	Steel X90CrMoV18	${39.2}_{-5.4}^{+7.8}$	${46.5}_{-3.2}^{+2.3}$
65 % Glycerol	AlTiN/CrN/Cr/…/CrN/Cr	${32.1}_{-5.8}^{+6.4}$	${\mathrm{48,7}}_{-2.1}^{+2.2}$
65 % Glycerol	AlN/CrN/…/AlN/CrN	${38.5}_{-4.6}^{+5.5}$	${\mathrm{45,2}}_{-2.4}^{+2.3}$
65 % Glycerol	CrN/AlN/…/CrN/AlN	${61.7}_{-4.8}^{+54}$	${\mathrm{24,5}}_{-1.4}^{+1.4}$
65 % Glycerol	SiO_2_	${53.1}_{-7.7}^{+8.4}$	${35.1}_{-2.9}^{+2.6}$
65 % Glycerol	Cr	${66.8}_{-5.4}^{+5.1}$	${34.9}_{-2.4}^{+1.7}$
99 % Glycerol	Steel X90CrMoV18	${46.3}_{-4.6}^{+4.8}$	${47.4}_{-1.1}^{+1.1}$
99 % Glycerol	AlTiN/CrN/Cr/…CrN/Cr	${42.7}_{-3.2}^{+4.1}$	${45.2}_{-1.3}^{+1.5}$
99 % Glycerol	AlN/CrN/…/AlN/CrN	${45.2}_{-3.7}^{+2.4}$	${47.4}_{-1.0}^{+1.4}$
99 % Glycerol	CrN/AlN/…/CrN/AlN	${69.8}_{-3.4}^{+2.8}$	${35.2}_{-1.1}^{+1.2}$
99 % Glycerol	SiO_2_	${62.3}_{-4.3}^{+3.8}$	${33.8}_{-1.3}^{+1.6}$
99 % Glycerol	Cr	${74.6}_{-3.6}^{+4.2}$	${47.4}_{-1.9}^{+1.1}$
Oil	Steel X90CrMoV18	${26.1}_{-5.9}^{+5.8}$	${31.2}_{-1.8}^{+1.6}$
Oil	AlTiN/CrN/Cr/…CrN/Cr	${12.4}_{-2.2}^{+3.4}$	${28.8}_{-1.4}^{+1.2}$
Oil	AlN/CrN/…/AlN/CrN	${20.6}_{-1.5}^{+1.8}$	${30.9}_{-1.2}^{+1.7}$
Oil	CrN/AlN/…/CrN/AlN	${54.6}_{-1.6}^{+2.1}$	${18.7}_{-1.9}^{+1.6}$
Oil	SiO_2_	${46.7}_{-1.1}^{+2.1}$	${26.6}_{-1.1}^{+1.3}$
Oil	Cr	${56.2}_{-2.7}^{+1.5}$	${25.1}_{-1.2}^{+1.2}$

## Results and discussion

In the internal combustion piston engine, during the movement of the piston inside the cylinder sleeve, there is a variable instantaneous speed of the moving kinematic pairs. This variation is due to the reciprocating motion of the piston. The piston ring set moves in a stochastic manner in the piston grooves, which makes it difficult to accurately determine the relative position of the moving surfaces of the piston and piston rings relative to the surface of the cylinder. However, thanks to advanced forecasting methods, we can determine this relative position with high probability. This assessment takes into account many factors, such as the dynamic viscosity of the lubricating oil, the detailed geometry of all major engine components, the crankshaft speed, and the prevailing thermodynamic conditions in the combustion chamber. The thickness of the oil film between the piston rings and the cylinder wall is not constant. It varies depending on many engine operating parameters, which has been highlighted in various studies, including^72^ and others. Using both simulation techniques and empirical data, engine researchers and designers can approximate the thickness of this oil film between specific engine components. It should be noted that the thinnest part of this film is usually between the uppermost ring of the piston and the cylinder wall. According to various studies, such as those carried out by Refs.^73,74^, this thickness oscillates between 0.1 and 15 μm. This variation depends on factors such as engine stroke phase, crankshaft speed, and various other parameters that determine engine load conditions. It is crucial to emphasize the importance of oil properties in this context. As confirmed by various studies, including^75,76^, oil characteristics have a large impact on key indicators such as hydrodynamic pressure. In addition, the nature of the oil also determines whether the engine operates in mixed or boundary friction conditions. Such considerations emphasize the necessity of choice proper oil formulation as this can directly affect engine efficiency, wear and overall performance.

In the tests, the measurement of the thickness of the lubricating film was carried out. Various loads were assumed for selected sets of sliders with applied coatings and lubricating fluid. The angle of inclination remained unchanged throughout the test. The name of the slider comes from the name of the coating. During the interferometric study, the number of fringes at different speeds was read and analyzed. In the test, the same number of evenly spaced fringes shifted in the given interferograms at different speeds means that the slider inclination remained unchanged at the time of testing, and no elastic deformations of the kinematic pair contact surfaces were observed. All tests were carried out under hydrodynamic lubrication conditions, reflecting the conditions of fluid friction between engine components. Experiments with 65 wt.% glycerol were carried out using various sliders with a constant load of 5 N and a constant slope of 1:1875. Like the slope plane of the ring relative to the surface of the cylinder for symmetric and asymmetric parabolic shapes. The use of interferometry in hydrodynamic lubrication provides valuable information that can help optimize lubrication processes, select the right lubricants, and understand wear resistance mechanisms.

**Figure 1:**
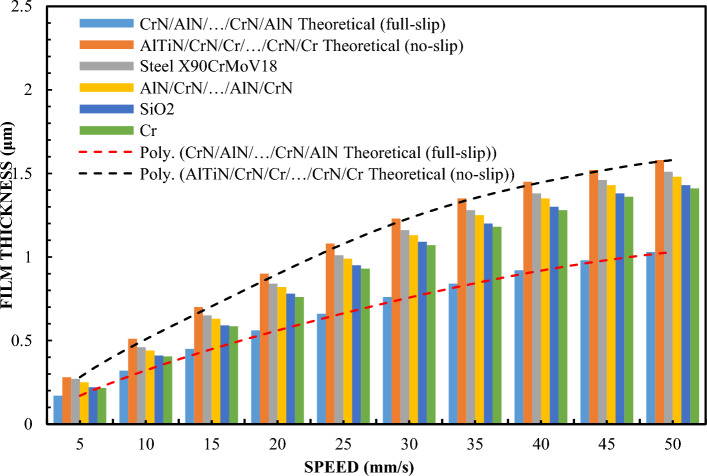
The thickness of the lubricating film layer depending on the speed of movement for 65% by weight of glycerol and the load W = 5 N.

Figure 1 shows the variation of the layer thickness depending on the velocity for a 65% sample of a glycerol solution. To better understand the issues of hydrophilicity and hydrophobicity, two theoretical curves of film thickness vs. velocity are additionally illustrated. Preparation of a stable 65% glycerin solution requires accurate measurement and mixing of pure glycerin with distilled water. It is important to ensure that the mixture is homogeneous and free of air bubbles. The glycerin solution tends to change these properties depending on the concentration. A drop test or a contact angle test can be used to determine these characteristics. In the study, also in order to organize the results, theoretical Reynolds equations were calculated on the basis of a full two-dimensional finite difference solution for surfaces covered with a lubricating film. The theoretical curve read under no-slip conditions corresponds to the classic Reynolds Eq. (1). Theoretical results for full slip conditions were calculated using an extended model of the Reynolds equation with boundary conditions corresponding to full slip, as in the case of Eq. (2)^77^. Equation (2) uses the critical stress slip model. In this model, stresses of this type are assumed to be zero. Comparing the terms of the equation on the right side of both equations, including one with boundary conditions for full slip (Eq. (2)), it was found to be only half of that with boundary conditions without slip. As a consequence of these findings and assumptions, it can be assumed that the theoretical thickness of the layer in boundary conditions of full slip is smaller. The results of this are shown in Fig. 1.

1 $$\frac{\partial }{\partial \mathrm{x}}\left({h}^{3}\frac{\partial \mathrm{p}}{\partial \mathrm{x}}\right)+\frac{\partial }{\partial \mathrm{y}}\left({h}^{3}\frac{\partial \mathrm{p}}{\partial \mathrm{y}}\right)=6u\eta \frac{dh}{dx}$$

2 $$\frac{\partial }{\partial \mathrm{x}}\left({h}^{3}\frac{\partial \mathrm{p}}{\partial \mathrm{x}}\right)+\frac{\partial }{\partial \mathrm{y}}\left({h}^{3}\frac{\partial \mathrm{p}}{\partial \mathrm{y}}\right)=3u\eta \frac{dh}{dx}.$$

X90CrMoV18 steel is one of the more advanced varieties of stainless steel known mainly for its excellent corrosion resistance and special mechanical properties. X90CrMoV18 steel is a martensitic steel. Its typical chemical composition is: carbon (C): 0.85–0.95%, chromium (Cr): 17.0–19.0%, molybdenum (Mo): 0.9-1.3%, vanadium (V): 0.07–0.12%. Other elements, such as silicon, manganese and phosphorus, are present in trace amounts. Thanks to its high chromium content, this steel has excellent corrosion resistance, making it ideal for use in chemically aggressive environments. Molybdenum and vanadium increase the hardness of the steel, making it abrasion resistant, while its martensitic nature allows high strength to be achieved by heat treatment. X90CrMoV18 steel can be hardened and tempered to obtain the optimum combination of hardness and strength. These properties make this steel suitable for use in the construction of piston engines exhaust.

The AlN/CrN/…/AlN/CrN coating is a multilayer composite that consists of alternating layers of aluminum nitride (AlN) and chromium nitride (CrN). Each of these layers contributes to the global properties of this advanced coating. Thanks to the combination of AlN and CrN, the coating has excellent resistance to high temperatures, which makes it ideal for applications that require thermal stability mainly for piston rings and pistons of internal combustion engines. The integrated CrN layers provide excellent hardness of the coating, which makes it highly resistant to abrasion and scratches, which is important during the running-in period, mainly of cylinder surfaces and piston rings. The AlN layers help improve the adhesion of the coating to various substrates while ensuring its integrity and cohesion under various conditions. This allows for further design of the properties of this composite in combination with other modern coatings with given properties, e.g. good wettability, good thermal resistance and good anti-friction properties.

The AlTiN/CrN/Cr/…/CrN/Cr coating consists of alternating layers of aluminum-titanium nitride (AlTiN), chromium nitride (CrN) and pure chromium (Cr). This unique multi-layer system translates into the specific properties of the composite. Aluminum Titanium Nitride (AlTiN) is known for its excellent resistance to high temperatures, making this coating ideal for applications in extreme thermal conditions. The combination of AlTiN and CrN provides the coating with exceptional hardness and abrasion resistance, which is the key to its durability. Chromium (Cr) layers improve the adhesion of the coating to various substrates and act as a corrosion barrier, providing additional corrosion protection. The AlTiN/CrN/Cr/…/CrN/Cr coating sets a new standard in the field of thin-film coatings, combining the excellent properties of aluminum-titanium nitride, chromium nitride and pure chromium. Its ability to work in harsh conditions and adapt to a wide range of industrial applications makes it one of the most promising materials in the field of surface engineering, in particular, it is highly promising in the application of highly mechanically and thermally loaded piston main components of the internal combustion engine.

The device's shoes made of X90CrMoV18 steel and AlN/CrN/…/AlN/CrN ensured a high layer thickness that coincided with the classical theory of hydrodynamic lubrication without slipping. The highest layer thickness results were also obtained for the AlTiN/CrN/Cr/…/CrN/Cr coating. It is a novel, complex multi-layer coating, created by the author in such a combination on the basis of theoretical considerations in order to obtain special wetting properties. The thickness of the formed lubricating film created by the slider covered with SiO2 and Cr was almost the same and slightly smaller than in the case of sliders AlTiN/CrN/Cr/…/CrN/Cr, AlN/CrN/…/AlN/CrN and X90CrMoV18 steel. The thickness of the lubricating film created by the CrN/AlN/…/CrN/AlN coated slider was the lowest. In this case, its change depending on the speed perfectly correlates with the theory of hydrodynamic lubrication under full slip conditions. This indicates that the molecular bonds of CrN/AlN/…/CrN/AlN and the lubricant are weak difference in test conditions was the surface roughness. Other parameters for all trials such as slider inclination, fluid parameters were the same.

The surface roughness of the slider with CrN/AlN/…/CrN/AlN coating is higher than for other coatings (Table 1). Nevertheless, the roughness of this coating is almost an order of magnitude less than the measured minimum layer thickness. Slightly lower roughness was obtained for X90CrMoV18 steel, AlTiN/CrN/Cr/…/CrN/Cr and AlN/CrN/…/AlN/CrN coatings. The Cr and SiO2 coatings had the lowest roughness. Nevertheless, this parameter did not significantly affect the oil film thickness for the X90CrMoV18, AlTiN/CrN/Cr/…/CrN/Cr and AlN/CrN/…/AlN/CrN steel coatings. Assuming these surface properties, it can be concluded that the small thickness of the lubricating film layer obtained from the CrN/AlN/…/CrN/AlN slider cannot result from high surface roughness. Otherwise, a higher roughness of the applied coating would increase the hydrodynamic effect leading to increased coating thickness. In this arrangement, the thickness of the lubricating film produced on the surface of the slider depends on the effects inter-surface and surface.

**Figure 2:**
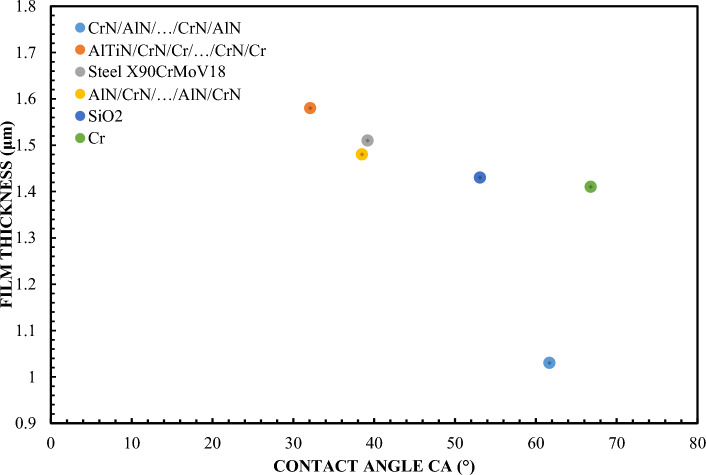
Correlation of layer thickness and contact angle (film thickness for the highest test values in accordance with Fig. 1)—65 % Glycerol.

Figure 2 shows the relationship between the thickness of the lubricating layer and the contact angle and CAH. These data provide important information about the interaction between the surface of the solid and the liquid that is distributed over it. The analysis of the accompanying graphs shows that the thickness of the lubricating layer decreases markedly as the contact angle increases. This is consistent with generally accepted theory in the field of tribology, which suggests that a larger contact angle usually indicates lower affiliation to the liquid, resulting in a thinner lubricating layer. For the materials adopted in the study, it can be stated that they behave in accordance with the general conceptual assumption. However, the Cr and SiO_2_ coatings are an exception. With these coatings, although the contact angle is the second largest among all the adopted slider variants, they generate a significant thickness of the lubricating layer. This may suggest that for these particular materials other factors such as such as surface chemistry or microstructure, can affect fluid interactions more significantly than contact angle alone. This discovery is not only fascinating, but also of crucial importance for engineers and scientists who strive to design coatings and materials with optimal lubricating properties. To fully understand these interactions and the intricate mechanisms behind them, further research and experiments are required that take into account a wide range of parameters and material properties.

**Figure 3:**
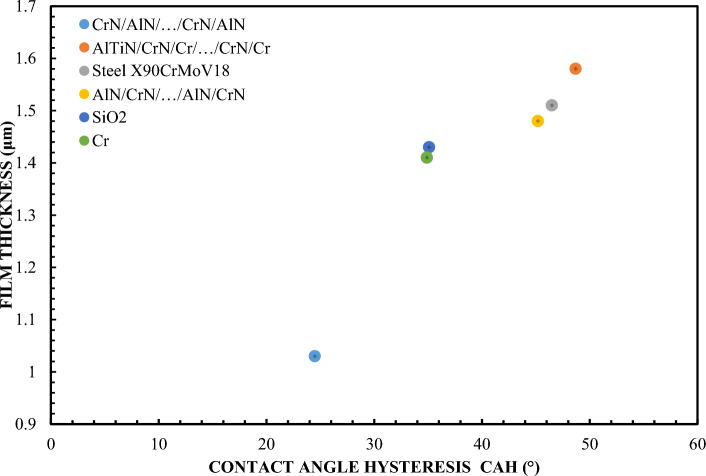
Correlation of layer thickness and contact angle hysteresis—65 % Glycerol.

Based on the data presented in Fig. 3, it can be hypothesized that the correlation between the thickness of the lubricating layer and the CAH (wet hysteresis angle) is significantly stronger than in the case of the contact angle (CA) alone. In the context of predicting the lubricating thickness for individual materials, CAH shows greater precision and predictability. The CAH waveform is more stable and linear compared to the CA waveform. This creates the potential for a better understanding of the interaction mechanisms between the liquid and the solid surface in the context of lubricating properties. During the analysis of Fig. 3, slight deviations of materials such as SiO_2_, Cr and CrN/AlN/…/CrN/AlN from the assumed approximation curve are noticeable. Such deviations may result from many factors, such as specific chemical properties of the material, surface structure or rheological properties of the liquid. For a more accurate approximation, a sixth polynomial was used for the contact angle (CA). degree, while for CAH it is a fourth degree polynomial. Such a mathematical method allows for a better mapping and understanding of the complex relationships between the analyzed parameters. In the future, further analysis may help pinpoint these correlations more precisely and provide scientists with key information for designing materials with optimal lubricating properties.

**Figure 4:**
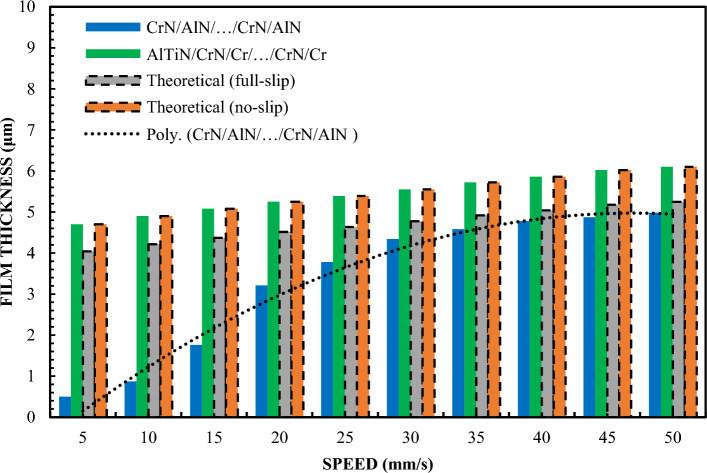
Thickness of the lubricating layer as a function of speed for 99% glycerin, with a load of W = 5 N.

**Figure 5:**
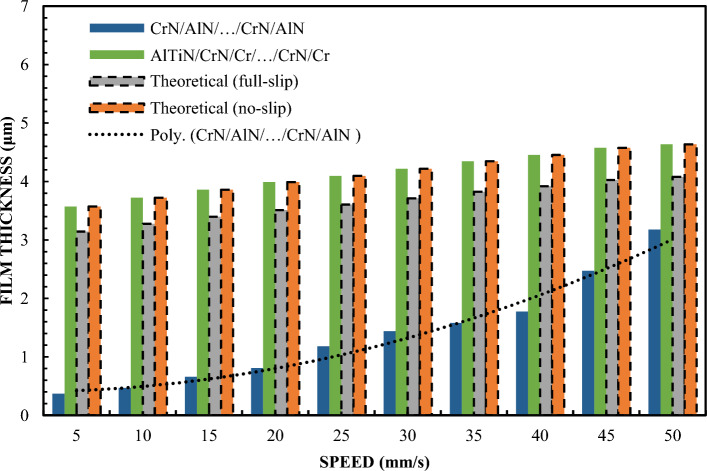
Thickness of the lubricating layer as a function of speed for 99% glycerin, with a load of W = 10 N.

Figure 4 shows the differences in the behavior of the CrN/AlN/…/CrN/AlN and AlTiN/CrN/Cr/…/CrN/Cr sliders in the context of creating a lubricating layer. Both types of sliders symbolize two extreme cases of the ability to form such a layer. To confirm these differences, tests were carried out using glycerin with a content of 99%. Changing the concentration of glycerin from 65% to 99% allowed to increase the viscosity. Tests were carried out for loads of 5 and 10 N at a slope of 1:1650. The results of these tests for both loads are shown in Figs. 4 and 5 for the same coatings. Tests have shown that the measurement results for the AlTiN/CrN/Cr/…/CrN/Cr coated sliders perfectly correlate with the theoretical anti-slip curves in the specified speed range for both loads. In turn, the thickness of the lubricating layer generated by the CrN/AlN/…/CrN/AlN slider is much smaller. Due to large measurement errors of interference images of the CrN/AlN/…/CrN/AlN slider at low speeds, Figs. 4 and 5 represent only the thickness of the lubricating layer measured at higher speeds, starting from 5 mm/s. Nevertheless, the minimum coating thickness shown in Figs. 4 and 5 is still about five times greater than the roughness of the CrN/AlN/…/CrN/AlN slider given in Table 1. Therefore, it can be assumed that there is no direct contact between the two mating surfaces selected kinematic pairs.

A significant reduction in the thickness of the lubricating layer for the CrN/AlN/…/CrN/AlN coating on the slider surface is undoubtedly related to the hydrophobic properties of this coating, which intensifies the repulsion of lubricant particles at low feed speeds. Such observations are in line with data available in the literature, where the hydrophobic properties of materials play a key role in interactions with liquids. As can be seen from the data presented in Table 3, the hysteresis of the contact angle of the CrN/AlN/…/CrN/AlN and AlTiN/CrN/Cr/…/CrN/Cr surfaces with 99% glycerin is 35.2° and 45.2°, respectively °. It is believed that the higher the CAH value, the greater the thickness of the lubricating layer. Figs. 4 and 5 reveal an intriguing phenomenon where the actual thickness of the lubricating layer obtained by the CrN/AlN/…/CrN/AlN coated slider is less than the theoretical full slip curves. To get a more complete picture of the influence of surface properties on the formation of the lubricating layer, tests have been carried out repeated with Olalphaolefin oil. This oil has a similar value of dynamic viscosity to glycerin with a content of 99%. However, the two substances differ in their polarity values. Olalphaolefin oil is a non-polar oil while glycerin is a polar substance. According to the data known from the publication, the polarity properties have a significant impact on the interaction with the surface and can affect the behavior of the lubricating film. Such an extension of the scope of research will enable a more accurate comparison and assessment of the possibility of influencing the oil film thickness in the operating conditions of a piston oil engine. Ultimately, these studies provide valuable information that can be used to optimize coatings in industrial applications where control of lubricant film thickness is crucial.

**Figure 6:**
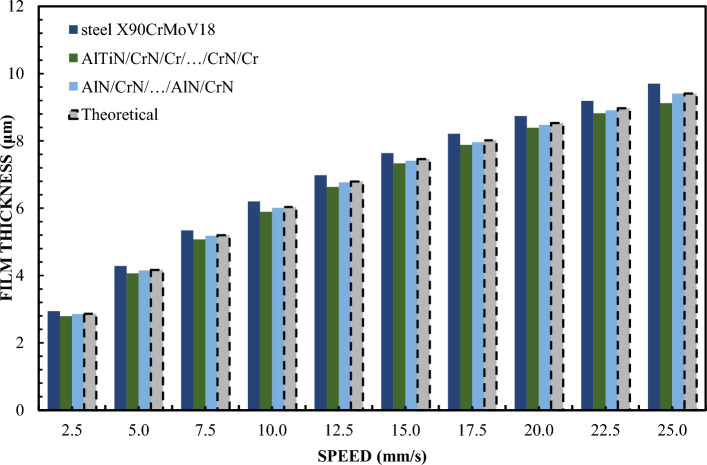
Lubrication film thickness for oil vs. speed for 5 N load.

**Figure 7:**
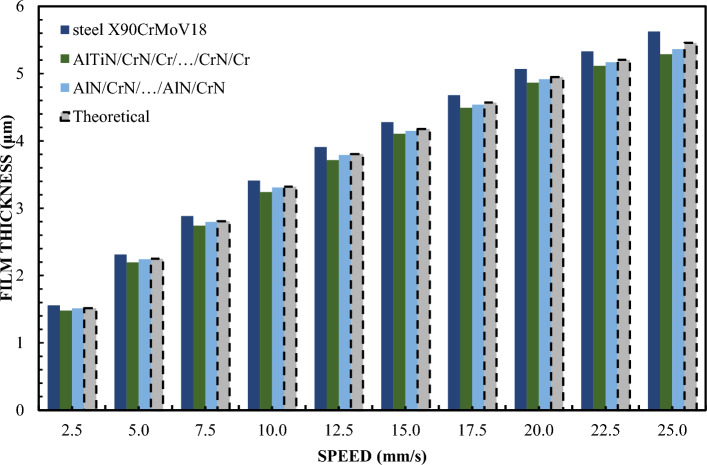
Lubrication film thickness for oil vs. speed for 10 N load.

Figures 6 and 7 show the variation in the thickness of the lubricating layer for oliphaolefin oil in relation to the speed of movement at a specific slope (deceleration: 1:1820) for loads of 5 and 10 N. In this experiment, all accepted materials were included. Sliders made of materials such as X90CrMoV18 steel, AlTiN/CrN/Cr/…CrN/Cr, AlN/CrN/…/AlN/CrN and CrN/AlN/…/CrN/AlN were tested under the same measurement conditions. It is interesting to note that these coatings show large variations in the contact angle CA, which are: 26.1°; 12.4°, 20.6°, 54.6°. However, for CAH, these values are respectively: 31.2°; 28.2°; 30.9°; 18.7°. The first three coatings show divergent CA values but have very similar CAH values. Therefore, it is necessary to precisely analyze which of these parameters better reflects the theoretical predictions of CA or CAH thickness. The courses of the film thickness distributions for CAH correlate well with classical theory of non-floating hydrodynamic lubrication. The difference in the contact angle between the material CrN/AlN/…/CrN/AlN and AlTiN/CrN/Cr/…/CrN/Cr is CA = 35.2°. In the case of sliders made of X90CrMoV18 steel and AlN/CrN/…/AlN/CrN, the difference is CA=5.5°. It is also interesting to note that the Cr coating shows a CA=56.2° and a CAH of 25.1°. The SiO2 coating, on the other hand, has a CA value lower by 7.9° compared to the CrN/AlN/…/CrN/AlN coating.

**Figure 8:**
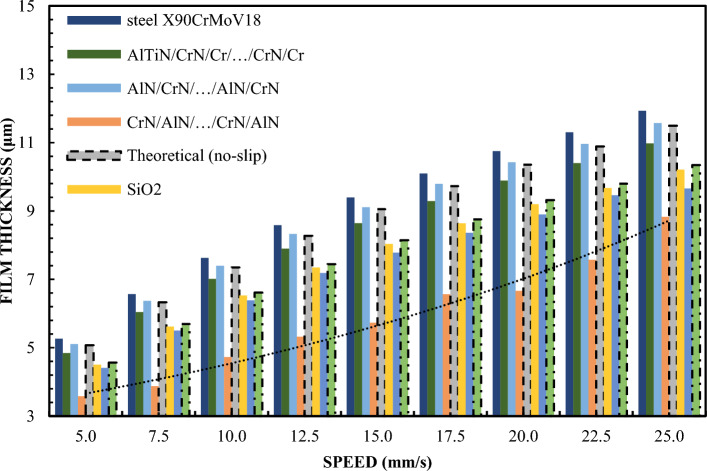
Lubrication film thickness for oil vs. speed for 2 N load.

**Figure 9:**
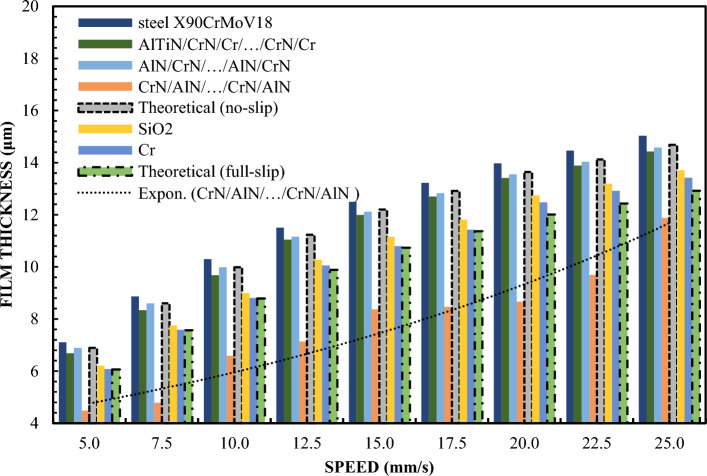
Lubrication film thickness for oil vs. speed for 1 N load.

Figures 8 and 9 show the thickness of the lubricating film layer for the oil produced by selected sliders at different speeds. The presented results clearly indicate that it is the CAH that more accurately reflects the relationship of the kinematic node for the effect of hydrodynamic lubrication produced by various sliding surfaces. This proves that the CAH parameter allows for a better determination of the conditions of hydrodynamic lubrication between the two surfaces. In this correlation, CA fares worse. In assessing the credibility of introducing the results of experimental research into mathematical models related to hydrodynamic lubrication, assessing hydrophobic and hydrophilic properties, this is an aspect of key importance. Therefore, CAH gives greater certainty in assessing the variability of the oil film thickness in the elements of the cooperating kinematic pairs of the engine. In contrast, CA shows greater deviations from the values of theoretical models and approximation functions. This analysis highlights the importance of considering the correct parameter when evaluating hydrodynamic lubrication conditions.

Test results for sliders covered with X90CrMoV18 steel, AlTiN/CrN/Cr/…CrN/Cr and AlN/CrN/…/AlN/CrN correlate perfectly with the classical theory of non-slip hydrodynamics for loads of both 1 and 2 N. On the other hand, the film thickness of the CrN/ AlN/…/CrN/AlN is significantly lower compared to the other sliders. The values of the film thickness in relation to the speed are characterized by a significant dispersion of values and inhomogeneity of the course. This coating is hydrophobic, while the other three are hydrophilic. Between them are the oil film thickness distributions for Cr and SiO_2_. However, they have a more even distribution of film thickness values with respect to the speed of the slider. Differences in the thickness of these layers resulting from differences in surfaces in this case correlate well with their CAH values, but not with the contact angle CA. In the author's opinion, this is the answer to the question of which parameter is more important when improving the model of the hydrodynamic theory lubrication. In addition, the results presented in Fig. 4.5, 8 and 9 indicate an incomprehensible phenomenon where the thickness of the film produced by the CrN/AlN/.../CrN/AlN slider with 99 wt. glycerol or oil is much lower than the theoretical full slip values. It is difficult to explain this phenomenon of the stochastic course of values for hydrophobic coatings. Comparing with the scientific literature, it can be concluded that the behavior of hydrophobic coatings under hydrodynamic lubrication conditions is a complex field that requires a multidimensional approach to research.

In the scientific work^63^, an in-depth analysis of the impact of the non-vetting, stationary surface of a sliding object on the hydrodynamic properties was carried out, using the model of critical shear stresses. Limit slip conditions were defined there: full slip (τc = 0), unidirectional slip (finite constant value τc applied to the entire static surface of the slider) and directional slip (slip or no slip and direction of slip depending on the local pressure gradient at the boundary). These studies were conducted on the basis of one-dimensional flow. It is interesting that analyzes of this type concerning two-dimensional flows are presented in Ref.^64^. They represent changes in the dimensionless bearing capacity W* where (${Wh }_{0}^{2}$/(Uη ${B}^{2}$ L) with respect to the dimensionless critical value of the shear stress τc* (h_0_ τc /Uη) for different inclinations of the rubbing pairs. Emphasizing the concept of shear on the slider surface, according to the results presented in Ref.^6^, is cru5cial importance for understanding hydrodynamic mechanisms in real conditions. It is worth noting that these studies complement other works in this field, creating a more complete picture of the impact of boundary conditions on hydrodynamic properties. In the context of practical applications, such research may be of key importance for the design of lubrication systems and the assessment of the durability of machine components.

High critical shear stress values τc*>1 correspond to non-slip conditions, and resistance curves approximate Reynolds load values. The ability to transfer the hydrodynamic load, i.e. to create a lubricating film, decreases when the limit slip begins on the stationary surface of the slider covered with any material. The disappearance of the hydrodynamic effect in the initial phase occurs very quickly with the increase in slip, which indicates a decrease in τc*. Soon this phenomenon is reduced to a minimum, and the ability to form a lubricating film increases again as τc* is further reduced. Such a description is particularly important in the case of correcting the slip values for various materials used for the components of kinematic pairs of internal combustion engines. This is the basic condition for the planned formation of the oil film in terms of surface coverage and the obtained thickness of the lubricating film.

Figure 1 shows that the thickness of the lubricating film layer created by the CrN/AlN/.../CrN/AlN slider with 65% wt. with the glycerol solution coincides with the theoretical full slip curve (i.e. τc = 0). This means that the critical shear stress on the surface of an EGC slide lubricated with a 65 wt. glycerin takes values leading to a significant reduction of the hydrodynamic effect, i.e. load support, as shown in Fig. 9. As a result, the thickness of the lubricating film layer produced by the CrN/AlN/.../CrN/AlN slider with 99% wt. of glycerin or oil is much less. Under these conditions, the direction of lubricant slip in the pre-surface area of the slider is towards the inlet, which results in reduced grease uptake and leads to a lower lubricant film thickness than under full slip conditions. The two glycerin samples have similar chemical properties but a big difference in dynamic viscosity. In this case, 99% wt. glycerin has a higher viscosity. Huge stresses shear 99% wt. of glycerin on the surface layer of the CrN/AlN/.../CrN/AlN slider material can be attributed to its higher dynamic viscosity compared to 65% by mass glycerin. The use of different mass percentages of glycerin results in different viscosity properties, which directly affects the ability to generate a stable lubricating film. Therefore, it is important to understand the influence of various factors, such as viscosity, on the behavior of the lubricant in practical applications.

Experiments performed on selected sliders with different surface materials illustrate the effect of the surface properties of different materials in terms of CA (contact angle) and CAH (contact angle hysteresis) on hydrodynamic lubrication. The ability to create a hydrodynamic lubricating film is related to the adhesive force between the liquid and the solid surface. This process takes place under conditions of fluid friction during the separation of the cylinder walls, piston rings and piston. Mixed friction conditions and the values of generated forces depending on operating conditions are not taken into account here. Lubricant molecules are only able to slide against any coating surface applied to an engine component only when these molecules overcome an energy barrier. Its value depends on the adhesion of lubricant particles and the coating. These parameters are specified using CAH and CA. The magnitude of the energy barrier depends on the interface properties of the coating and lubricant. Additionally, it is worth emphasizing that optimizing the surface properties of materials, especially in terms of CA and CAH, can lead to significant benefits in terms of hydrodynamic lubrication performance. This allows you to reduce the wear of engine components and extend their life.

In the article^45^, based on the basic principles of thermodynamics, the energy barrier equation was derived. It is expressed in CA (contact angle) and CAH (contact angle hysteresis):

3 $$E=\frac{\gamma R}{{2}^\frac{7}{3}}{\left(CAH\right)}^{2}f\left(\theta \right),$$

where:

4 $$f\left(\theta \right)=\frac{{\left(1+cos\theta \right)}^{2}}{{\left(1-cos\theta \right)}^{1/6}{\left(2+cos\theta \right)}^{4/3}}.$$

The final form of the equation depends on the specific assumptions and approaches used in the derivation. Although the contact angle and the hysteresis of the contact angle are important parameters characterizing the interaction between the liquid and the solid surface, their exact influence on the adhesion and sliding mechanisms is complicated and depends on many factors, such as the type of lubricant, temperature, pressure and surface properties of the material.

Energy barrier calculations can be performed using the parameters θ and CAH. However, the value of this parameter does not change significantly in the range from 20° to 140°. The function f(θ) remains practically constant over this range. A significant part of the value of the energy barrier is determined by the CAH. In the context of materials used in the construction of kinematic components of internal combustion engines, it is assumed that a contact angle exceeding 90° indicates the hydrophobic properties of the material surface. Therefore, taking into account the operating conditions of combustion engines and the maximum piston speeds, which are temporarily much higher than in the test, the hydrophobic coating characteristics are not necessarily worse in terms of the formation of a lubricating layer than in the case of hydrophilic coatings. However, a more in-depth study of both hydrophobic and hydrophilic properties under real operating conditions is required for a full assessment.

Based on the formula (3), it was established that the lower the CAH value, the lower the energy barrier value. The basic factor of this relationship is the molecular interaction between the lubricating liquid and the coating surface. Limit slip occurs only above CA 140°. The slip length also increases above this value. A number of studies on research methodology to assess the validity of the use of CA and CAH are presented in Refs.^75–80^. According to the author, the research methodology adopted and the obtained results confirm the significant potential of the theory of material selection in terms of CA and CAH in industrial applications in internal combustion engines, not only to achieve durability of these assemblies and low wear, but also to significantly reduce hydrodynamic friction losses occurring between main components of reciprocating internal combustion engines.

In order to more precisely analyze this contact angle and the contact angle hysteresis, new samples were introduced, which are a combination of a TiN coating deposited on a SiO_2_ base coating. These coatings are highly hydrophobic. The main objective of the study was to assess the usefulness of individual CA and CAH measurements depending on the grain size and roughness of coatings on surface layers with comparable hardness. This became the answer to how CA and CAH behave depending on the morphology of the coatings.

The contact angle and the hysteresis of this angle were compared with the size distribution based on scanning electron microscopy (qualitative) and atomic force microscopy images (quantitative). They were used to describe the topography and structure of liquid distribution on the TiN coating on a SiO_2_ substrate depending on the reaction parameters, primarily temperature and film deposition time.

The contact angle and hysteresis were recorded using a CCD detector for visualization acquisition. Image analyzes were performed using the CAM2008 program (KSV Instruments^®^). A drop of ultrapure water with a volume of 15 μl (with a resistivity exceeding 18.2 MΩ⋅cm) was used. The measurement procedure for each drop was repeated 10 times, and then the average value of the contact angle was determined. Corrected angle values were obtained by mathematical correction using the Young/Laplace model^81^. For visual characterization of the TiN coating and examination of the topography, a scanning electron microscope (SEM) model Quanta 250FEG from FEI, operating at an acceleration voltage of 20 kV, was used, and images were recorded using secondary electrons (SE). To determine the grain size distribution, SEM micrograph analysis was performed using image analysis tools in ImageJ^®^. Thanks to this, it is possible to assess the proportion of surface area occupied by individual grains relation to the entire tested surface. In order to study in detail the topography of the layer's surface and determine its roughness, AFM images were used using the AFM system from Bruker, operating at a room temperature of 20 °C.

**Figure 10:**
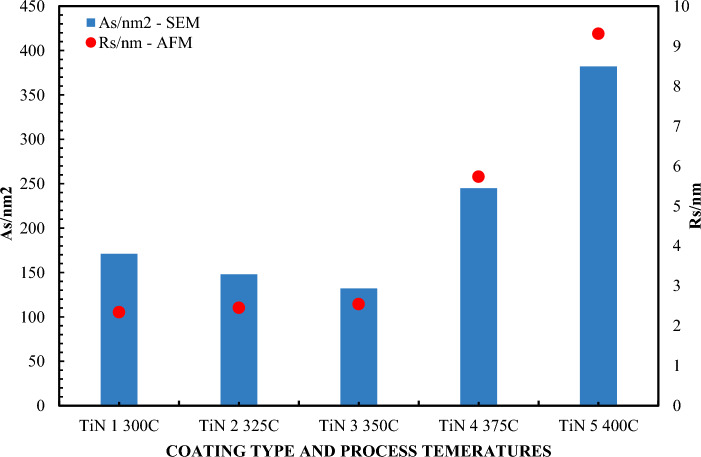
Histograms of the size distribution of As and Rs. for TiN coatings.

**Figure 11:**
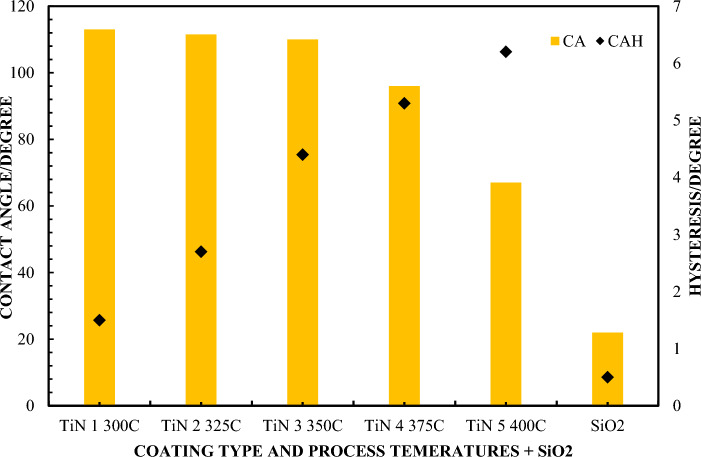
Measurements of the CA contact angle and CAH hysteresis for various types of TiN samples depending on the deposition temperature.

Figure 10 shows the average Ra values for selected samples obtained using SEM and AFM. This finding makes it possible to state that the reduction in hydrophobicity does not result from modifications in the nature of surface wetting, but from transformations that are related to unevenness and grain size. A reference to the contact angle and contact angle hysteresis depending on different surface structures and morphologies of the surface layers is shown in Fig. 11. In the context of thermal variability, the observations indicate an interesting phenomenon: while the CA contact angle values are decrementing, the COH hysteresis increases, which suggests antagonistic behavior of these two parameters. Correlatively, the average interfacial area occupied by micro-irregularities and the grain structure on the surface showed increasing trends with increasing temperature, as shown in Fig. 10. The standard deviation for the measurements in Fig. 10 does not exceed 3.8%, and for measurements from Fig. 11 does not exceed 5.3%.

**Figure 12:**
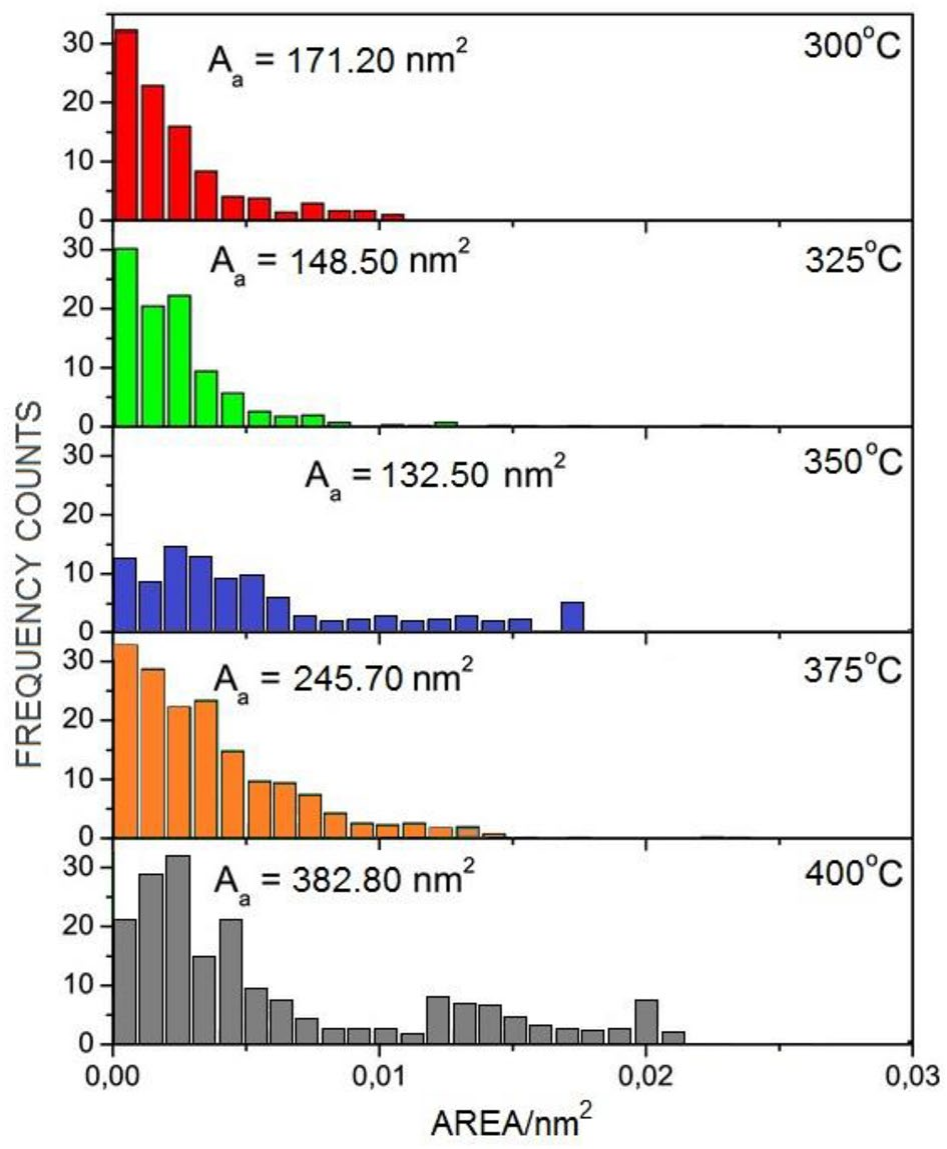
Histograms of grain size distribution for TiN coatings.

Analyzing morphological parameters, such as roughness and average surface area occupied by grains, an increasing trend was observed depending on temperature. These parameters present variations analogous to the grain size distribution, as shown in the histograms in Fig. 12. Such observations are of key importance in the context of the methodology for extrapolating data regarding the irregularities or irregularities of the surface of the examined layers. Based on the analyzes performed, we can postulate that the contact angle decremented in proportion to the increase in surface irregularities of the TiN coating. In opposition to this phenomenon, contact angle hysteresis demonstrated anticorrelation behavior. However, both of these characteristics are related to the morphology of the surface layers of the coating. Therefore, it can be concluded that regardless of the surface treatment technology, the key parameter determining the intensity of hydrophobic and hydrophilic features of coatings is surface morphology. Moreover, it is worth noting that the accuracy and assessment of surface wettability by using the CA and CAH parameters with the advantage of the reliability of one of them is the surface topography. It depends on which parameter is better suited for assessing the wettability of CA or CAH. There is no clear answer, although based on the presented results it can be said that CAH works in most cases and its effectiveness in assessing the severity of hydrophilic or hydrophobic features is greater. All tested samples showed lower hydrophilicity (higher contact angle) than the SiO_2_ base material. These results indicate that TiN is highly hydrophobic compared to SiO_2_. However, the grain size distribution and the CA action angle and the CAH hysteresis change with the deposition temperature. This work does not analyze the coating application technology, but the influence of grains and roughness on the possible practical use of CA and CAH to assess the wettability of surfaces. hydrophobic and hydrophilic. This means that larger grains are associated with greater roughness and a smaller contact angle for the hydrophobic surface. Some articles mention the influence of surface roughness and heterogeneity as an important factor influencing the value of the contact angle. This effect is related to the contact angle hysteresis.

## Conclusions

In reciprocating internal combustion engines, oil film formation is dependent on many engine operating factors, both macro and micro component geometry, fluid properties and raw materials. Currently, the latest trend is to analyze the potential of designing the thickness of the oil film and coating the sliding surfaces of the rings, piston and cylinder with an oil film using the effects of hydrophilic and hydrophobic coatings.

The influence of these parameters on the oil film profile and the wetting ability of the kinematic pair materials surface is complicated to evaluate in variable conditions. There are two interface parameters: the contact angle and the contact angle hysteresis, the effectiveness of which in assessing these phenomena has been compared in terms of their interdependence with the hydrodynamic effect of lubrication. Several coating materials with different hydrophilic-hydrophobic properties, with different CA and CAH parameters, and three liquids, both polar and hydrophobic, were used in the tests. and non-polar, which gave contact angles ranging from 12° to 75°.

Moreover, in the context of coatings with larger grain granules, the CA contact angle decreased, while the CAH hysteresis of this angle increased. With respect to coatings with lower roughness (smooth surfaces), the contact angle was more elevated. The relationship between grain roughness and non-uniformity, contact angle CA and CAH hysteresis is a strong indicator that these measurements can be used to classify the structure of thin-layer anti-wear coatings used in piston combustion engines.

In the analysis, in order to predict the thickness of the oil film, it was found that the key parameter is the hysteresis of the contact angle CAH, which more adequately reflects the phenomena of oil film formation than the contact angle CA in the context of hydrophilic and hydrophobic characteristics. The results of these analyzes also correlate with the basic theory of thermodynamics. It was found that the critical shear stresses constituting the limit slip barrier depend on the dynamic viscosity of the lubricant. This discovery will open the door for future researchers to a deeper understanding of the hydrophilic and hydrophobic properties of coatings in the context of oil film formation and design, which have not been considered so far. This is crucial due to the constant striving to reduce engine mechanical friction losses while maintaining the durability of kinematic assemblies.

## Data Availability

All data generated or analyzed during this study are included in this published article.

## List of symbols

R: Radius of spherical liquid drop on solid surface
E: Potential energy barrier
B: Breadth of slider bearing (in x direction)
U: The speed of the puck in a given direction
L: Length of slider bearing (in y direction)
p: Fluid pressure
h: Film thickness
$\eta$: Dynamic viscosity of lubricant
τ_c_: Critical shear stress for slip
x: Shift coordinate
y: Transverse coordinate of the shift
