# Author Correction: The concept of the contact angle in the process of oil film formation in internal combustion piston engines

**DOI:** 10.1038/s41598-023-48949-x

**Published:** 2023-12-19

**Authors:** Piotr Wróblewski, Stanisław Kachel

**Affiliations:** 1 Faculty of Engineering, University of Technology and Economics H. Chodkowska in Warsaw, Jutrzenki 135, 02-231 Warsaw, Poland; 2 Faculty of Mechatronics, Armament and Aerospace of the Military University of Technology, ul. gen. Sylwestra Kaliskiego 2, 00-908 Warsaw 46, Poland

Correction to: *Scientific Reports* 10.1038/s41598-023-47763-9, published online 24 November 2023

The original version of this Article contained an error in Affiliation 2, which was incorrectly given as ‘Faculty of Mechatronics, Institute of Aviation Technology, Division of Aircraft Construction and Operation, Armament and Aerospace of the Military University of Technology, ul. gen. Sylwestra Kaliskiego 2, 00-908 Warsaw 46, Poland’. The correct affiliation is listed below:

Faculty of Mechatronics, Armament and Aerospace of the Military University of Technology, ul. gen. Sylwestra Kaliskiego 2, 00-908 Warsaw 46, Poland.

The original Article has been corrected.

